# A strength reliability calculation method of tip relief gears

**DOI:** 10.1371/journal.pone.0348272

**Published:** 2026-05-14

**Authors:** Hongbing Wang, Jun Xiao, Lairong Yin, Bo Hu, Huihua Liu

**Affiliations:** 1 Hunan Provincial Key Laboratory of Safety Design and Reliability Technology for Engineering Vehicle, Changsha University of Science and Technology, Changsha, P. R. China; 2 Mechanical Industry Key Laboratory of Medium and Small Sized Joints in Robots, Guangdong Desheng Intelligent Technology Co., Ltd, Foshan, P. R. China; 3 Beijing Engineering Research Center of Precision Measurement Technology and Instruments, Beijing University of Technology, Beijing, P. R. China; Beijing Institute of Technology, CHINA

## Abstract

The gear tip relief affects the root bending stress and the contact pressure, which affects the strength reliability of the gear system. In this work, based on the proposed tip relief model and the mesh model of the gear pair, incorporating the gear’s contact strength and bending strength models together with the stress–strength interference theory, a strength reliability model of tip relief gears was established by assuming that both the stress and strength follow a normal distribution. The results indicate that the tip relief reduces the meshing impact and increases the strength reliability. Then, the effects of the modification length, the modification height, and the applied load on the gear strength reliability were analyzed. It was found that with the increase in gear modification length and the modification height, both the contact and bending strength reliability at the engaging-in and the recession points decrease compared to the standard gears. Additionally, an increasing of the load also results in a decline in gear strength reliability. Therefore, the rational control of the gear loading and the appropriate selection of modification length and modification height are of great significance in reducing meshing impact while ensuring the strength reliability of gears.

## 1. Introduction

Gear drive is one of the most commonly applied forms of mechanical transmission due to its constant transmission ratio, high power capacity, high efficiency, and low noise. It is widely used in various power transmission systems, including automotive transmissions, aerospace vehicles, and industrial gear reducers [1]. During the service life of the gears, the mating surfaces are subjected to the repeated contact pressure, leading to pitting which degrades the transmission quality and even causes the contact fatigue failure. Under the heavy-loaded conditions, cracks often initiate at the gear root, resulting in tooth breakage and bending fatigue failure [2]. The contact strength and bending strength failures reduce the smoothness and the precision of the transmission, in severe cases, may cause equipment shutdowns or accidents, posing serious risks to system safety and reliability. Gear tooth profile modification typically implemented to mitigate the errors caused by the elastic deformation and the manufacturing errors, is commonly used to reduce the meshing impact, vibration and noise [3]. However, the influence of tooth profile modification on stress distribution and gear strength reliability remains insufficiently investigated. Therefore, it is necessary to investigate the reliability of the tooth profile modified gear systems under both the contact and the bending failure modes to facilitates the evaluation of system health, enhances the safety margins and the service life.

Reliability is defined as the ability of a product to perform its intended function under specified conditions for a designated period of time. When measured probabilistically, reliability is referred to as reliability index or reliability probability [4]. In early gear design, empirical formulas and safety factor methods were primarily used. Rao [5] was among the first to introduce the concept of system reliability into the structural reliability analysis and design of gear systems. By treating allowable stress as a normally distributed random variable, the reliability of planetary gear systems under two failure modes: root bending and surface contact fatigue is investigated. Subsequent researchers began to assess failure probabilities using stress–strength interference models [6, 7], and gradually incorporated random variables. Monte Carlo simulations based on sensitivity analysis were employed to evaluate the reliability of contact and bending fatigue strength. Peng et al. [8] compared a Monte Carlo-based finite element method (FEM) using a first-order second-moment method with a stochastic FEM (SFEM) based on a second-order third-moment model, accounting for multiple sources of uncertainty, such as material properties, dimensional tolerances, and loading conditions, thereby improving fatigue life prediction accuracy and robustness. With the deepening of research, attention has shifted toward the correlation among multiple failure modes and the dynamic reliability analysis of gear systems [9–13]. Salvatore et al. [14, 15] studied dynamic reliability with respect to the influence of dynamic loads on components; however, their analysis did not account for the time-dependent relationship between strength and loading. Cazuguel M et al. [16] investigated dynamic reliability problems involving nonlinear behavior. Li [17] proposed a dynamic fatigue reliability model for gears incorporating failure correlation and strength degradation, using a combination of the Copula function and the Gamma process. To enhance modeling and optimization efficiency, recent studies have integrated intelligent algorithms and surrogate models into gear reliability design [18, 19]. Li [20] used ANSYS finite element software to develop a finite element model for a meshing spur gear pair and performed strength analysis. A polynomial response surface surrogate model was then used to evaluate the system’s reliability and sensitivity.

Strength failure of gears is one of the primary factors affecting the reliability of transmission systems. The dominant failure modes include tooth surface wear and root fracture. Numerous researchers have investigated the contact and bending strength of gears. Early studies on contact pressure were derived from the Hertzian theory, treating the meshing of involute gears as the contact between two cylindrical rollers. The laid the theoretical foundation for gear surface contact strength calculations. The early calculation methods for bending stress were based on parabolic equal-strength cantilever beam models, from which the well-known Lewis formula was derived. Subsequently, Merritt [21] refined the Lewis formula by incorporating the effects of radial load components. With the advancement of computational technologies, researchers increasingly adopted FEM to perform numerical stress analysis [22, 23]. Li et al. [24] proposed a three-dimensional finite element method for accurately calculating the bending and contact strength of thin-walled gears under high-speed conditions. Hwang et al. [25] compared FEM-based contact pressure results of spur and helical gears with those obtained using AGMA contact pressure formulas and concluded that FEM typically predicted higher gear strength. Furthermore, many scholars have integrated finite element analysis with design of experiments, mathematical programming, and other numerical techniques to propose new methods for predicting and estimating contact and bending stresses in various types of gears [26–29]. Shi et al. [30] presented a method for analyzing the reliability of tooth surface contact strength, taking into account several types of profile errors. They combined experimental design techniques with FEM to sample contact pressure values and applied Weibull distribution for fitting and analysis. Raptis et al. [31] employed photo-elastic tests to compare the maximum bending stress at the gear root with FEM results and found that the error between the two increased with the number of gear teeth, yet remained within acceptable limits.

In traditional gear design, standard tooth profiles are prone to meshing impact or localized stress concentration during the initial or final stages of engagement. Tip relief, which involves intentionally removing material from the tooth tip, changes the stress distribution on the tooth surface, impacting both the contact and bending strength of the gears. Researchers have proposed various relief curves, including linear, parabolic, and exponential types, which have been optimized through FEM, experimental data, and dynamic simulation to achieve optimal meshing performance under different load and speed conditions [32–35]. Tip relief modification significantly influences the dynamic response of gear transmission systems [36]. Time-domain and frequency-domain analyses show that profile modification effectively reduces transmission error, vibration and noise, and is especially critical in high-speed and heavy-load gear systems [37, 38]. Zhou et al. [39] proposed a two-stage tip relief curve to enhance gear meshing performance by minimizing the range and amplitude of transmission error and reducing vibration displacement and noise, thus improving operational characteristics. Furthermore, investigating stress distribution in modified gears is essential for determining appropriate modification parameters [40]. Maper et al. [41] analyzed the stress distribution of tip-relieved spur gears using ANSYS, derived a bending stress formula, and introduced four expressions to estimate the contact and bending stress of tip-relieved gears. Wang et al. [42] treated tip relief as a form of profile manufacturing error, derived the error along the line of action, and developed models to compute load-sharing factors and contact pressure for modified profiles. In recent years, intelligent algorithms such as machine learning, genetic algorithms, and multi-objective optimization have been increasingly applied in tip relief design to quickly identify optimal modification parameters and improve design efficiency [43, 44].

In summary, traditional design methods based on safety factors can no longer meet the requirements of high-performance gear system for the stable load-carrying capacity and the high reliability. At present, most studies adopt the stress-strength interference theory and utilize FEM to model and analyze the strength reliability of gears. While existing studies on tip-relieved gears mainly address stress analysis before and after modification, meshing dynamics, and the influence of modification parameters on meshing performance, limited work has been conducted on the strength reliability of tip-relieved gears. Since gear strength reliability is closely related to stress distribution, although profile modification can improve localized stress states and dynamic behavior, its overall impact on system reliability remains uncertain. Moreover, the influence of modification parameters on gear strength exhibits strong nonlinearity and uncertainty. Without a systematic reliability analysis, improper modification could introduce new strength failure risks. Therefore, this study focuses on the gear system with tip relief, based on the finite element theory and the stress-strength interference model, a reliability evaluation model is established considering both the contact and the bending strength failure modes. The effects of the modification parameters and the loading conditions on stress distribution and strength reliability of the gears are investigated to elucidate the relationship between the profile modification and the gear reliability, providing the theoretical guidance for extending the service life of modified gear system.

## 2. Strength calculation model of tip relief gears

### 2.1 Tip relief of gears

**Fig 1:**
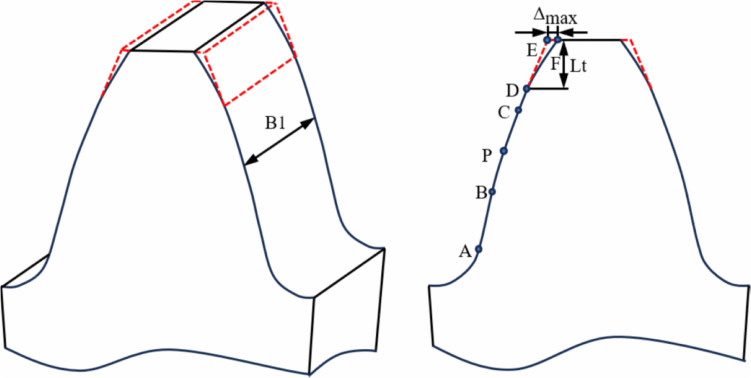
Tip relief of spur gear.

The Fig 1 illustrates the tip relief principle of a spur gear. In Fig 1, point P is the pitch point, point A is the engaging-in point, and point E is the recession point. Points B and C represent the lowest and highest points of single tooth contact (LPSTC and HPSTC), respectively. Point D marks the start of the tip relief, point E is the end point before modification (between the addendum circle and the involute profile), and point F is the end point after modification. Key parameters for tip relief include the type of modification curve, the maximum modification quantity Δ_max_ (usually call the modification height), and the modification length *L*_*t*_. The modification length is defined by the starting point of the tip relief. When the modification begins at the HPSTC during single-tooth contact, it is called long-modification. When it starts between the addendum circle of the tooth and the HPSTC during single-tooth contact, it is called short-modification.

The tip relief is typically performed using a power function curve and it can be expressed as follows:


$$\Delta_{i}=\Delta_{max}(\frac{L_{i}}{L_{t}}{)}^{s}$$ (1)

In Eq (1), $L_{i}$ denotes the difference between the radius of the base circle at an arbitrary modification point on the involute and the radius at the modification starting point D, with $L_{i}$∈ [0,$L_{t}$]. In engineering practice, the modification index S is typically chosen as 1, 1.5, or 2, where S=1 represents linear modification and S=2 corresponds to parabolic modification.

In this study, a parabolic tip relief curve is adopted. With recent advancements in gear machining equipment, parabolic modification has become increasingly common. Compared to linear modification, the parabolic curve provides a smoother transition at the addendum region, which more effectively reduces impact phenomena during gear meshing.

### 2.2 Gear strength modeling

**Fig 2:**
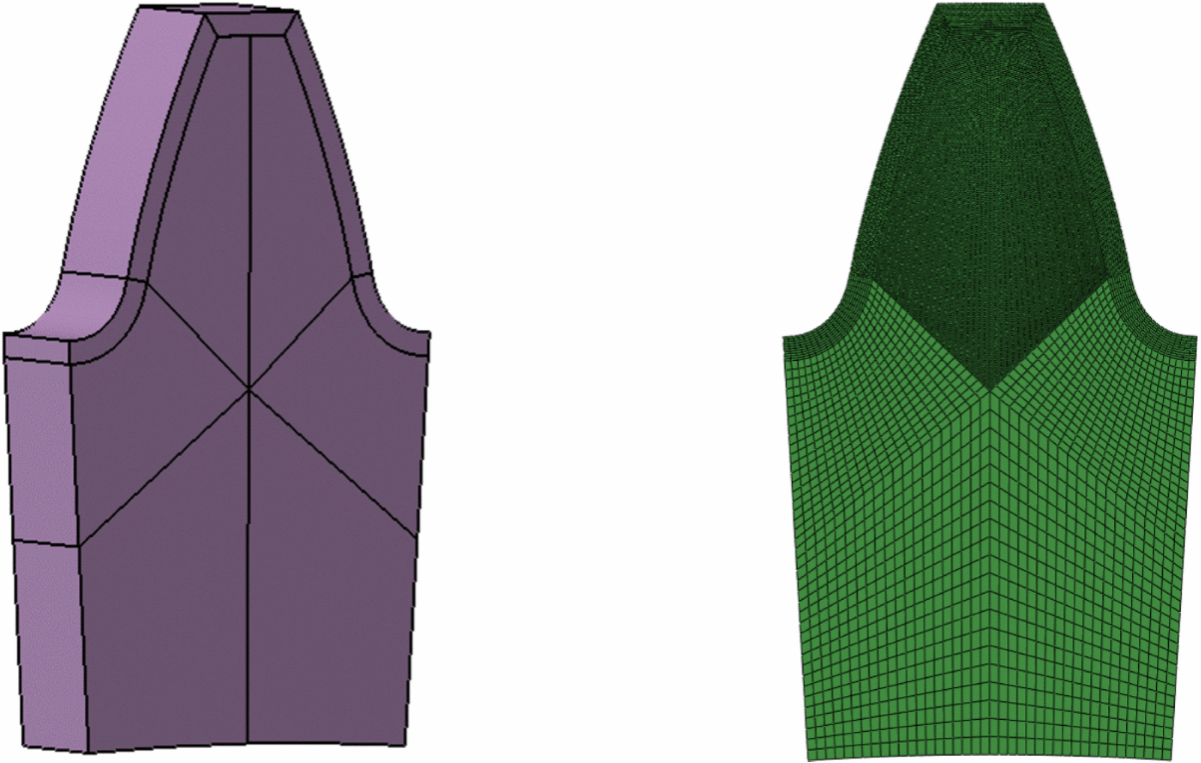
Single-tooth geometry and mesh generation model.

The bending strength and the contact strength of the gears are mainly determined by the root bending stress and the tooth surface contact pressure, which can be obtained through the contact analysis of the gear pair. Assuming that the gear center planes lie in the same plane and that shaft deformation is negligible during meshing, the profiles curve equation of the profile modified gears were established by the generating method [42]. To balance computational speed and accuracy, this paper adopts a two-dimensional gear finite element contact model. The element mesh is divided using plane strain quadrilateral elements (CPE4R), and appropriate mesh refinement is performed on the involute segment. The single-tooth geometry and the meshed model are shown in Fig 2.

**Fig 3:**
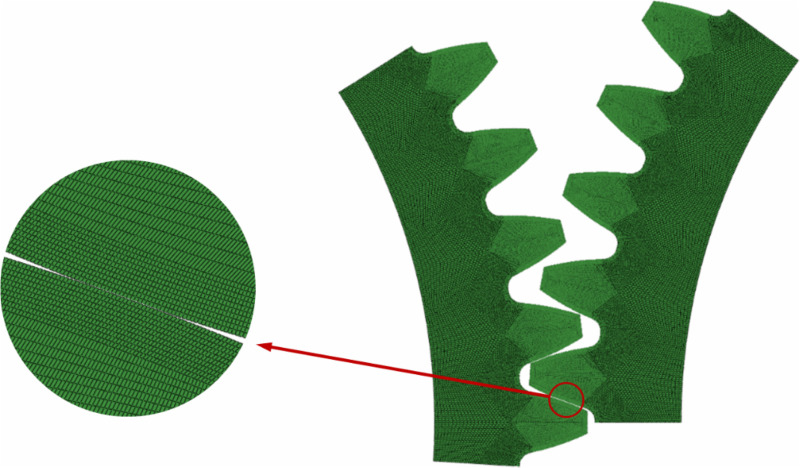
Contact model based on finite element method.

In general, the spur gears engage with at most two pairs of teeth simultaneously. Therefore, constructing a full finite element model of the entire gear would result in excessive computational resource consumption due to the presence of non-meshing teeth, leading to reduced simulation efficiency. To balance computational efficiency with modeling accuracy, a five-tooth gear segment was created using a circular array to simulate the meshing process. The configuration ensures that the target tooth undergoes a complete meshing cycle, including both engaging-in and recession. The stress value of the middle (third) tooth of the driving gear during meshing is recorded, and the maximum value among them is taken as the final simulation result for the group. The finite element five-tooth assembly diagram is shown in Fig 3. Using the contact model of the spur gears, the strength models for both the standard and the tip relief gears can be constructed.

After completing the aforementioned mesh division and assembly steps, further preprocessing of the finite element model is required, and the relevant steps are as follows:

#### 1. Definition of analysis steps.

To capture the full loading history of the gear meshing process, a quasi-static distributed loading approach was employed. The simulation steps were defined as follows:

Initial Step: All degrees of freedom (DOFs) of both gears were constrained to ensure convergence at the start of the simulation.

Step 1: The driven gear’s DOFs were fixed, and a small rotational displacement was applied to the driving gear to initiate contact between the gear pairs and allow the load to be smoothly transferred to the driven gear.

Step 2: The rotational DOF about the Z-axis of the driven gear was released, while the driving gear remained fully constrained. A clockwise torque was gradually applied to the driven gear until it reached the predefined magnitude by the end of the step.

Step 3: The rotational DOF of the driving gear about the Z-axis was released, and a counterclockwise rotational displacement was applied. To ensure numerical convergence and accuracy, the maximum time increment was limited to 0.01, with a total step time of 1. The meshing process primarily occurs in the step, during which the required output variables were collected.

#### 2. Contact and boundary condition setup.

In ABAQUS, contact properties are defined in terms of tangential and normal behaviors. As frictional forces were not considered in the analysis, the tangential behavior was set to “frictionless.” For the normal behavior, a “hard” contact formulation was used, allowing for separation after contact. Given the relative sliding that occurs between gear tooth surfaces during meshing, the “finite sliding” formulation was selected for the contact interaction.To simulate the fixed-axis rotation of the gear pair, rigid coupling constraints were applied using reference points. Reference points were created at the center axes of the driving and driven gears, respectively, and were rigidly coupled to the inner surfaces of the gear center holes. Rotational displacements were then prescribed at these reference points to control gear movement and contact progression. The setup ensured that the selected single tooth completed a full meshing cycle.

#### 3. Load application.

During meshing, gears are primarily subjected to the driving torque of the active gear and the resisting torque of the driven gear. To realistically simulate the loading condition, the torque on the driving gear was converted using the transmission ratio and equivalently applied to the reference point of the driven gear. A rotational displacement with the same direction was then applied to the driving gear to emulate the meshing transmission process. Both gears were constrained such that their motion was restricted to rotation about their own Z-axes only.

#### 4. Postprocessing.

After completing the model setup—including material properties, contact definitions, load application, and boundary constraints—the gear meshing angle was fine-tuned, and the mesh density on the contact surfaces and regions was refined iteratively until convergence was achieved. Leveraging the powerful computational capabilities of the ABAQUS solver, stress distributions along the theoretical tooth surface and root regions were obtained and visualized using color-contoured plots on the solid model. The stress evolution during the meshing process was analyzed by inspecting the visual stress contour maps and extracting XY data to generate stress output curves.

Using the method described above, strength models for both standard and tip relief gears were constructed, and postprocessing was performed to obtain the stress variation curves of a single tooth throughout a complete meshing cycle.

## 3. Gear strength reliability model

In mechanical reliability engineering, particularly during the initial design phase, the normal distribution is the most commonly used and effective model for describing variables composed of numerous independent, minor random factors (such as machining dimensional errors, material property fluctuations, and random components of loads). It provides a solid and widely recognized starting point for probability assessment under conditions of limited data. Factors related to gear contact pressure include external load F, gear width b, pitch circle diameter d, and various correction coefficients, all of which are random variables. When each random factor has a minor effect, these random variables can be approximated as following a normal distribution. If all these factors follow a normal distribution, gear contact pressure can be considered to follow a normal distribution; and gear bending stress can also be considered to follow a normal distribution, similarly. Furthermore, based on the stress distribution diagram of gears obtained from finite element model, it is evident that the working stress of gears has both concentration and symmetry. Therefore, the contact pressure and bending stress of gears can be treated as normally distributed. Consequently, understanding the normal distribution patterns of various stresses in gears aids further reliability analysis.

### 3.1 Reliability model

**Fig 4:**
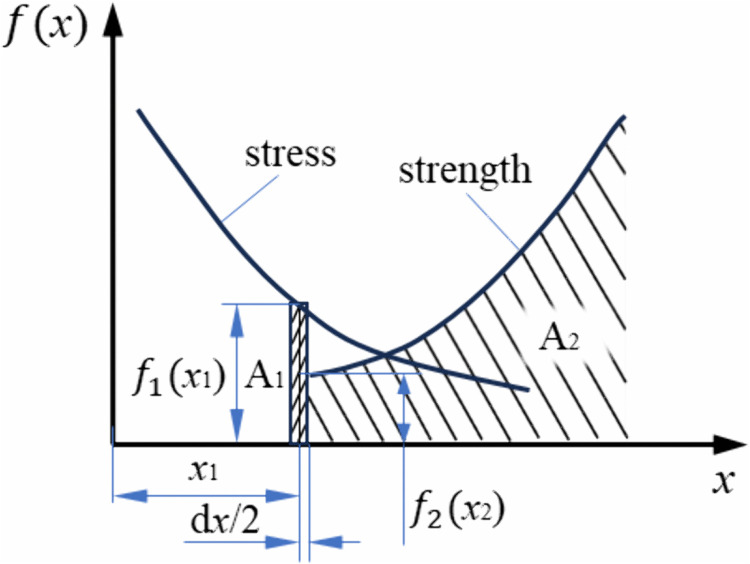
Stress-strength dynamic probability model.

Generally, the internal force per unit area of a part or product under external forces such as pressure or torque is called stress, denoted as X_1_, with its probability density represented by $f(x_{\text{1}})$. The ability of a part or product to resist permanent deformation and fracture is referred to as strength, denoted as X_2_, with its probability density represented by $f(x_{\text{2}})$. If stress is X_1_ and strength is X_2_, regardless of the distribution they follow, when stress exceeds strength (X_1_ > X_2_), the structure will fail; when strength exceeds stress (X_2_ > X_1_), the structure is reliable. Therefore, under the stress-strength interference model, the reliability of a part or product’s strength is the R = P (X_2_ > X_1_), as shown in Fig 4.

In Fig 4, the probability of stress within the interval dx/2 around a random point $x_{\text{1}}$ on the x-axis is represented by the shaded area A_1_. The probability that the strength exceeds the stress is indicated by the shaded area A_2_. The probability that the value of x falls within the $x_{\text{1}}\pm dx/\text{2}$ and the strength exceeds the stress is A_1_ × A_2_. For the entire stress distribution, the probability that the strength exceeds the stress, which is the reliability *R*. The above parameters can be represented by Eqs. (2), (3), and (4).


$$A_{\text{1}}=P\left(x_{\text{1}}-dx/2<X_{\text{1}}<x_{\text{1}}+dx/2\right)=f_{\text{1}}\left(x_{\text{1}}\right)dx_{\text{1}}$$ (2)

$$A_{\text{2}}=P\left(X_{\text{2}}>X_{\text{1}}\right)=\int_{x_{\text{1}}}^{\infty }f_{\text{2}}\left(x_{\text{2}}\right)dx_{\text{2}}$$ (3)

$$R=A_{\text{1}}\times A_{\text{2}}=\int_{-\infty }^{\infty }f_{\text{1}}\left(x_{\text{1}}\right)\left[\int_{x_{\text{1}}}^{\infty }f_{\text{2}}\left(x_{\text{2}}\right)dx_{\text{2}}\right]dx_{\text{1}}$$ (4)

When the working stress of a component X_1_ and its strength X_2_ both follow a normal distribution, Their probability density functions are given by Eqs. (5) and (6).


$$f_{\text{1}}\left(x_{\text{1}}\right)=\frac{\text{1}}{\sigma_{\text{1}}\sqrt{2\pi }}exp\left[-\frac{\text{1}}{\text{2}}{\left(\frac{x_{\text{1}}-\mu_{\text{1}}}{\sigma_{\text{1}}}\right)}^{\text{2}}\right]$$ (5)

$$f_{\text{2}}\left(x_{\text{2}}\right)=\frac{\text{1}}{\sigma_{\text{2}}\sqrt{2\pi }}exp\left[-\frac{\text{1}}{\text{2}}{\left(\frac{x_{\text{2}}-\mu_{\text{2}}}{\sigma_{\text{2}}}\right)}^{\text{2}}\right]$$ (6)

Where: $\mu_{\text{1}}$,$\;\mu_{\text{2}}$ represent the means of stress and strength, respectively, while $\sigma_{\text{1}}$,${\;\sigma }_{\text{2}}$ represent their standard deviations. Let Y = X_2_-X_1_; then Y also follows a normal distribution, $\text{Y}\sim N\left(\mu_{\text{y}},\overset{\text{2}}{\underset{\text{y}}{\sigma }}\right)$, with its probability density function given by:


$$f\left(y\right)=\frac{\text{1}}{\sigma_{y}\sqrt{2\pi }}exp\left[-\frac{\text{1}}{\text{2}}{\left(\frac{y-\mu_{y}}{\sigma_{y}}\right)}^{\text{2}}\right]$$ (7)

Where: $\mu_{y}=\mu_{\text{2}}-\mu_{\text{1}}$, $\sigma_{y}=\sqrt{\overset{\text{2}}{\underset{\text{2}}{\sigma }}+\overset{\text{2}}{\underset{\text{1}}{\sigma }}}$.

According to the stress intensity interference model, when the strength exceeds the stress (Y > 0), the product is safe and reliable. The reliability *R* is:


$$R=\int_{\text{0}}^{\infty }f\left(y\right)dy=\int_{\text{0}}^{\infty }\frac{\text{1}}{\sigma_{y}\sqrt{2\pi }}exp\left[-\frac{\text{1}}{\text{2}}{\left(\frac{y-\mu_{y}}{\sigma_{y}}\right)}^{\text{2}}\right]dy$$ (8)

Let $z=\left({y-\mu }_{y}\right)/\sigma_{y}$, after standard normalization is:


$$R=\frac{\text{1}}{\sqrt{2\pi }}\int_{z}^{\infty }exp\left[-{\frac{z}{\text{2}}}^{\text{2}}\right]dz=1-\phi \left(z\right)$$ (9)

In the above formula, once the standard normal variable *z* is determined, one can refer to the standard normal probability integral table to obtain the reliability calculation value.

### 3.2 Gear strength reliability model

In traditional reliability models for series gear systems, the overall system reliability is evaluated based on the weakest component principle. The approach assumes that the various failure modes contributing to gear failure are linearly dependent. Specifically, the contact strength or fatigue safety margin 𝐺_1_, and the bending strength or fatigue safety margin 𝐺_2_, are considered linearly correlated (Safety margin G is defined as strength minus stress). Let 𝐹_1_ denote the failure probability due to contact strength or contact fatigue, and 𝐹_2_ denote the failure probability due to bending strength or bending fatigue. The corresponding reliabilities under each failure mode are defined as $R_{H}$ and $R_{F}$, respectively. In the conventional reliability model, the total system reliability 𝑅 is calculated as the product of the reliabilities under each independent failure mode, as expressed by:


$$R=R_{H}\times R_{F}$$ (10)

However, in practical scenarios, the correlation coefficient ρ≠1, but lies within the range 0<ρ<1.The actual gear system reliability model is an extension of the traditional series system model, with the key distinction being the presence of statistical correlation between the two failure modes.

#### 1. Reliability analysis of gear tooth surface contact strength.

According to previous analysis, the contact pressure on the gear tooth surface follows a normal distribution. Therefore, to determine the contact strength reliability of the gear, it is essential to identify the probability distribution of the contact strength of the tooth surface. Literature indicates that the contact strength of the gear surface also follows a normal distribution. The assessment primarily considers whether the structural integrity of the gear component can withstand the maximum operational load, while neglecting the influence of stress concentration, size factors, and other secondary effects. For gear materials with Brinell hardness (HB) in the range of 150–620, the mean value and standard deviation of the contact strength limit are as follows:


$${\overset{―}{\mu }}_{rH}=2.76HB-68.95$$ (11)

$$\sigma_{rH}=0.14HB$$ (12)

According to the associated equation, the reliability index of the gear under the tooth surface contact strength mode can be expressed as:


$$Z_{R_{H}}=\frac{{\overset{―}{\mu }}_{rH}-{\overset{―}{\mu }}_{sH}}{\sqrt{\overset{\text{2}}{\underset{rH}{\sigma }}+\overset{\text{2}}{\underset{sH}{\sigma }}}}$$ (13)

#### 2. Reliability analysis of gear tooth root bending strength.

Under the action of external loads, tensile stress is generated at the tooth root of the gear. Therefore, the bending strength of the gear is primarily determined by the material’s maximum tensile capacity. For materials with a Brinell hardness (HB) in the range of 200–450, the static bending strength can be estimated using the following empirical formula:


$${\overset{―}{\mu }}_{rF}=3.41HB$$ (14)

$$\sigma_{rF}=0.14HB$$ (15)

According to the associated equation, the reliability index of the gear under the bending strength mode can be expressed as:


$$Z_{R_{F}}=\frac{{\overset{―}{\mu }}_{rF}-{\overset{―}{\mu }}_{sF}}{\sqrt{\overset{\text{2}}{\underset{rF}{\sigma }}+\overset{\text{2}}{\underset{sF}{\sigma }}}}$$ (16)

#### 3. Reliability calculation based on contact and bending strength failure modes.

Based on the previously calculated failure probabilities for contact and bending static strength, the corresponding reliability indices can be used to determine the individual reliability index for each failure mode. These indexes represent the probability that the gear will not fail under contact or bending conditions, respectively. By integrating the two failure modes, the overall reliability of the gear system under combined contact and bending strength failure modes can be derived.

The comprehensive reliability index of the gear is given by:


$$Z_{R}=-\phi^{-1}\left[1-\phi \left(Z_{R_{H}}\right)\phi \left(Z_{R_{F}}\right)\right]$$ (17)

The reliability of the gear based on contact and bending strength failure modes is:


$$R=\int_{-\infty }^{\infty }{\left[\phi \left(\frac{z+\sqrt{\rho t}}{\sqrt{1+\rho }}\right)\right]}^{\text{2}}\varphi \left(t\right)dt$$ (18)

$$\rho =\frac{cov\left(G_{\text{1}},G_{\text{2}}\right)}{\sqrt{D\left(G_{\text{1}}\right)D\left(G_{\text{2}}\right)}}=\frac{E\left(G_{\text{1}},G_{\text{2}}\right)-E\left(G_{\text{1}}\right)E\left(G_{\text{2}}\right)}{\sigma_{G_{\text{1}}}\sigma_{G_{\text{2}}}}$$ (19)

In Eq (18) and Eq (19), *ρ* represents the correlation coefficient between the safety margins of contact strength and bending strength of the gear. In the gear transmission system, although the tooth surface contact fatigue and the tooth root bending fatigue belong to different failure mechanisms, they are not independent of each other. Its strong correlation is mainly due to its common material properties and common load history. Therefore, ignoring this strong correlation (assuming *ρ* = 0) in system reliability modelling will overestimate the reliability of the system; on the contrary, if it is regarded as completely correlated (*ρ* = 1), it may be too conservative. Based on the current material properties and working condition, the correlation coefficient *ρ* is 0.9293.

## 4. Results and discussion

### 4.1 Validation of the gear strength model

#### 4.1.1 Contact strength validation.

**Fig 5:**
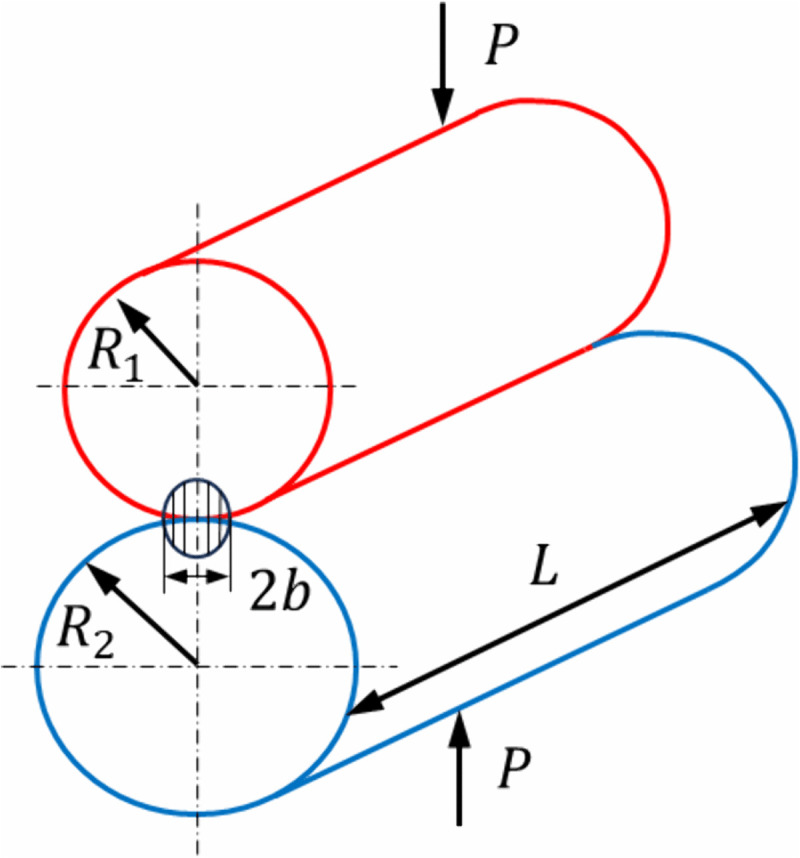
Hertzian contact model.


The calculation of contact pressure on gear teeth in industrial standards across various countries is still fundamentally based on Hertzian theory. As shown in Fig 5, the contact model between the two gears can be approximated as line contact between two cylindrical bodies prior to deformation. When subjected to an external load P, both cylinders undergo elastic deformation, resulting in a rectangular contact area, as illustrated by the shaded region in Fig 5. Assuming the total width of the contact area is 2*b*, the contact half-width b and the maximum contact pressure $P_{max}$ can be expressed as:

$$b=\sqrt{\frac{4P(\frac{1-\overset{\text{2}}{\underset{\text{1}}{\nu }}}{E_{\text{1}}}+\frac{1-\overset{\text{2}}{\underset{\text{2}}{\nu }}}{E_{\text{2}}})}{\pi L(\frac{\text{1}}{R_{\text{1}}}+\frac{\text{1}}{R_{\text{2}}})}}$$ (20)

$$P_{max}=\sqrt{\frac{P(\frac{\text{1}}{R_{\text{1}}}+\frac{\text{1}}{R_{\text{2}}})}{\pi L(\frac{1-\overset{\text{2}}{\underset{\text{1}}{\nu }}}{E_{\text{1}}}+\frac{1-\overset{\text{2}}{\underset{\text{2}}{\nu }}}{E_{\text{2}}})}}$$ (21)

In Eq (20) and Eq (21), *L* denotes the effective contact length between the two elastic cylinders. *R*_*i*_、*E*_*i*_、*ν*_*i*_ (*i* = 1,2) represent the contact radius, elastic modulus, and Poisson’s ratio of each elastic cylinder, respectively.

**Fig 6:**
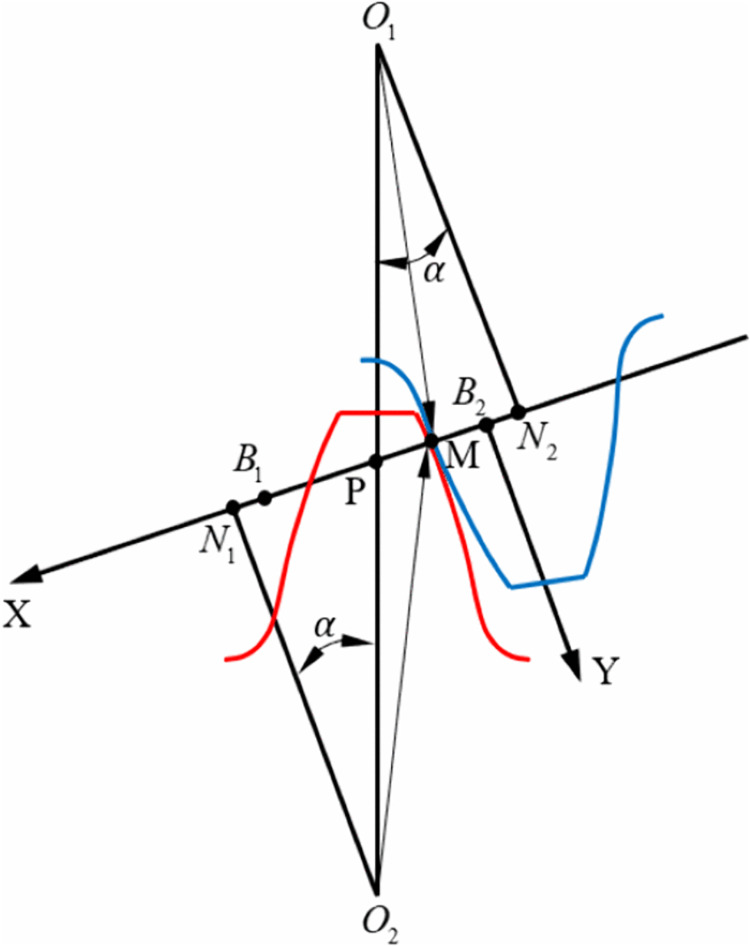
Meshing process of the gear pair.

The meshing process of the gear pair is illustrated in Fig 6. Engaging-in begins at point *B*_2_, where the root of the driving gear contacts the tip of the driven gear, and continues until recession at point *B*_1_, where the tip of the driving gear contacts the root of the driven gear. During the meshing process, the gear pair contact can be approximated as the contact between two cylinders with time-varying radii, whose centers are located at points *N*_1_ and *N*_2_, respectively.

For spur gear pairs with a contact ratio between 1 and 2, the meshing process of a single gear pair transitions from double-tooth contact to single-tooth contact and back to double-tooth contact. Accordingly, the load on each tooth pair varies dynamically throughout the meshing cycle. To accurately determine the contact pressure distribution on the gear flank, it is necessary to calculate the load borne by each tooth pair using the load distribution factor *l*. When only one pair of teeth is in contact, that pair bears the entire load, thus *l* = 1. During double-tooth meshing, the load is shared between two adjacent tooth pairs. Assuming equal elastic deformation, the load distribution between the teeth can be determined based on the stiffness of each contact pair, leading to the following relationship:


$$\Delta s=F_{\text{1}}/k_{\text{1}}=F_{\text{2}}/k_{\text{2}}$$ (22)

In Eq. (22), $F_{i}$ and $k_{i}$ represent the load borne by the *i*-th tooth pair and its time-varying meshing stiffness, respectively, where *i* = 1,2.

Moreover,


$$F=F_{\text{1}}+F_{\text{2}}$$ (23)

$$F=\frac{T}{r_{\text{1}}cos\alpha }$$ (24)

In Eqs. (23) and (24), F denotes the total load transmitted through gear meshing, T is the input torque applied to the driving gear, $r_{\text{1}}$ is the pitch circle radius of the driving gear, and α is the pressure angle.

From Eqs. (22) and (23), it can be derived that:


$$\left\{ \begin{array}{c} @l@l_{\text{1}}=k_{\text{1}}/(k_{\text{1}}+k_{\text{2}})\\ l_{\text{2}}=k_{\text{2}}/(k_{\text{1}}+k_{\text{2}}) \end{array}\right.$$ (25)

In Eq. (25), *l*_*i*_ (*i* = 1,2) represents the load distribution coefficient for the *i*-th tooth pair during double-tooth meshing.

Therefore, the load borne by each tooth pair can be expressed as:


$$F_{i}=F\cdot l_{i}$$ (26)

At any given moment during gear meshing, the contact pressure on the tooth surface can be calculated using Hertzian contact theory. At the time, the applied load P is equal to *F*_i_, the load borne by the *i*-th tooth pair. By establishing a coordinate system as shown in Fig 6, the contact pressure at any point M along the line of action can be expressed as:


$$\left\{ \begin{array}{c} @l@R_{\text{1}}=N_{\text{1}}B_{\text{2}}+x_{M}\\ R_{\text{2}}=N_{\text{2}}B_{\text{2}}-x_{M} \end{array}\right.$$ (27)

In the Eq. (27), $x_{M}$ denotes the *x*-coordinate of an arbitrary meshing point M in the coordinate system XB_2_Y.

**Fig 7:**
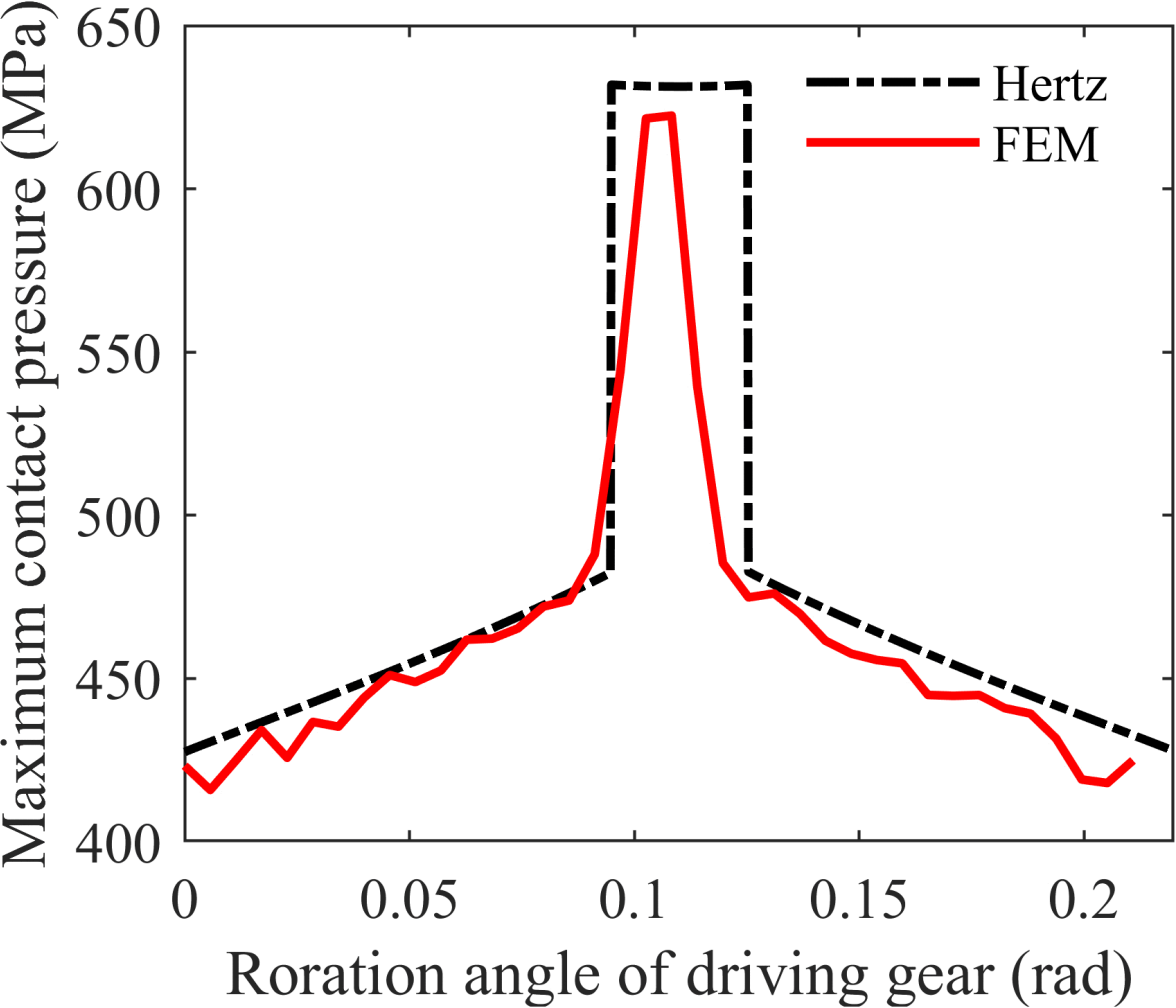
Comparison between hertzian contact pressure and finite element model results.

To verify the accuracy of the proposed contact model, a gear contact model was established in the finite element analysis software ABAQUS using the gear pair parameters listed in Table 1. A plane strain model was selected, and CPE4R (4-node bilinear plane strain quadrilateral) elements were used for meshing. When the element width in the contact region does not exceed one-third of the Hertzian contact half-width, the accuracy of finite element contact pressure results is considered high and sufficient for research purposes. For the gear model in the study, the Hertzian contact half-width is calculated as 0.1394 mm. Taking into account computational performance and time efficiency, the finite element mesh size within the contact region was set to 0.0394 mm. The curves of the maximum contact pressure obtained by Hertzian theory and by finite element simulation, as functions of rotation angle over one meshing cycle, are shown in Fig 7.

**Table 1:** Standard gear parameters.

Parameters	Value	Parameters	Value
Number of teeth	Z1 = 50, Z2 = 50	Input speed	n1=50 r/min
Module	m = 3 mm	Load torque	T_in_=300 N·m
Pressure angle	α=20°	Modulus of elasticity	E_1_ = 206GPa, E_2_ = 206GPa
Face width	B = 30 mm	Poisson’s ratio	u_1_=0.3, u_2_=0.3

It can be observed that the contact pressure curve obtained from the finite element analysis of the gear model closely matches the curve calculated using Hertzian contact theory, with both exhibiting similar variation trends throughout the meshing cycle. It also shows that due to the limited number of mesh grids of the FEM, the contact pressure distribution between the tooth surfaces will have some small fluctuations. On the other hand, the contact pressure values at the points of engaging-in and recession obtained from the finite element model are slightly lower than those predicted by Hertzian theory. In addition, the single-tooth meshing area is narrower in the finite element results, and the maximum contact pressure within the region is also lower compared to the Hertzian-based values. The main reasons for the above phenomenon are as follows: the calculation of the contact pressure for gear pairs based on the Hertzian contact theory mainly involves two steps. Firstly, calculate the load sharing factor between teeth based on the time-varying meshing stiffness of the gears. Then, consider the gear pair as the contact of two cylindrical bodies with time-varying radii, and calculate the contact pressure based on the Hertzian contact theory. When calculating the time-varying meshing stiffness of the gears, the single tooth and the concentrated force are used, without considering the elastic deformation of adjacent teeth contact during meshing. Therefore, there will be a strict division of single-tooth contact and double-tooth contact, and the single-tooth contact occupies a relative longer meshing time. While in the finite element calculation model, calculates the contact pressure based on the actual meshing process of the gear pair, including the contact of adjacent teeth, and the single-tooth contact area occupies a relatively shorter meshing time. When the meshing process is about to enter the single-tooth contact area geometrically, the teeth involved in the meshing at the previous moment will not be completely separated immediately due to the elastic deformation. The adjacent gear pairs still maintain contact and share a part of the load. This results in a narrowing of the single-tooth meshing area and a decrease in the peak pressure.

**Fig 8:**
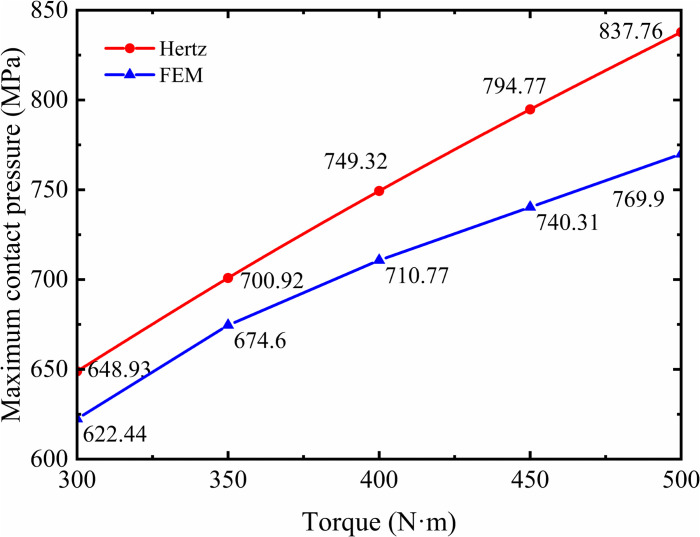
Comparison of Hertzian and FEM maximum contact pressures under varying torques.

To further investigate the discrepancy between the maximum contact pressure calculated by the Hertzian model and the finite element model, five sets of data under torque loads ranging from 300 N·m to 500 N·m are compared, as illustrated in Fig 8.

It can be observed that within the torque range of 300 N·m to 500 N·m, the variation curves of the maximum contact pressure per meshing cycle obtained from both Hertzian contact theory and finite element model remain largely consistent. The error between the two methods gradually increases with torque, but remains within the range of approximately 4.1% to 8.1%. The confirms the reliability of the finite element model in predicting contact pressure during gear meshing.

#### 4.1.2 Bending strength validation.

Currently, the mainstream method for calculating gear bending strength in traditional design is based on continuous improvements to the Lewis formula. The approach comprehensively accounts for the effects of load, contact ratio, and tooth profile on the calculated results, and incorporates various correction factors to enhance accuracy.

**Fig 9:**
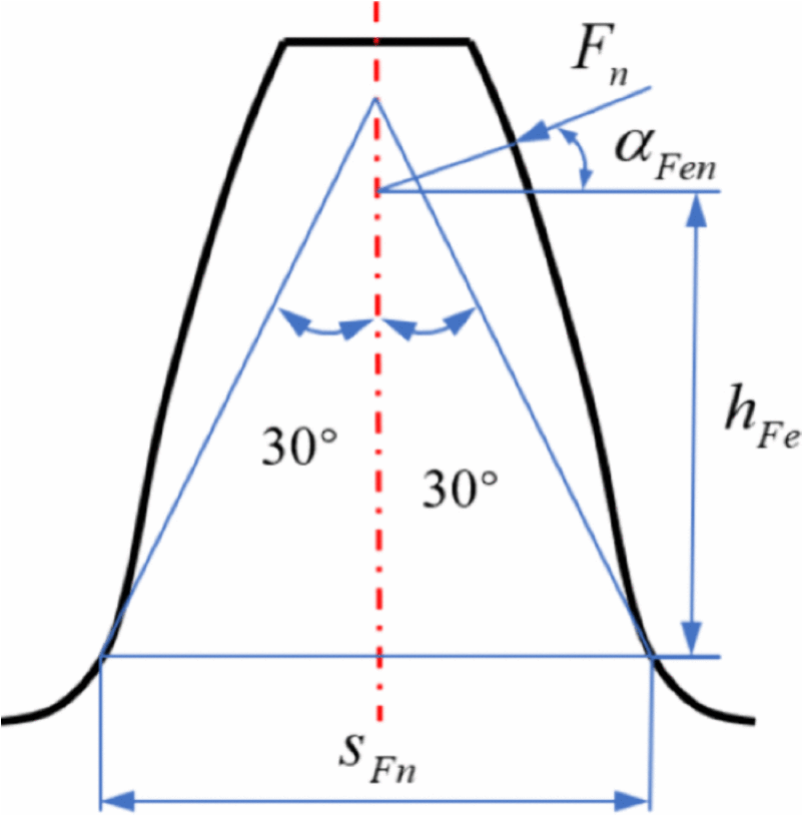
30° tangent method.

The primary theoretical issue in studying the bending strength of the gear tooth root is the identification of the critical section. When the bending stress at the root exceeds the allowable bending strength of the material, tooth fracture may occur. The fracture typically initiates at the critical section. For spur gears, failure is most likely to occur under single-tooth meshing when the load acts at the highest point of the specified region. Once the critical section is identified, the most failure-prone area is known; ensuring sufficient strength at the location effectively guarantees the structural integrity of the entire gear. In the study, the 30-degree section method is used to determine the position of the critical section, as shown in Fig 9. A line is drawn at an angle of 30° from the gear symmetry center and made tangent to the fillet curve at the tooth root. The cross-section that passes through both tangency points and is parallel to the gear axis is defined as the critical section.

When the gear is in a single-tooth meshing state, the load is borne entirely by one pair of teeth, resulting in relatively high bending stress. The bending stress reaches its theoretical maximum when the load is applied at the highest point within the single-tooth meshing area. Gear bending strength verification is typically conducted by evaluating the stress at the tooth root. Most theoretical models and formulas for calculating root bending strength are based on the standard ISO 6336−3: 2019, which specifies that the tooth root bending fatigue strength should be evaluated using the tensile-side stress at the tooth root. While the radial component of the normal load increases compressive force and reduces tensile stress, leading to higher compressive-side bending stress, strength verification must still use the tensile-side value. The is because fatigue crack initiation is more sensitive to tensile stress. Therefore, in the following calculations, the compressive-side bending stress will not be considered. The classical formula for calculating the gear tooth root bending strength is given in Eq. (28).


$$\sigma =\frac{F_{t}}{bm}Y_{F}Y_{S}K_{A}K_{V}K_{F\beta }K_{F\alpha }$$ (28)

In the equation: ${\text{F}}_{\text{t}}$ is the nominal tangential load at the pitch circle, b is the face width of the gear, m is the module, ${\text{Y}}_{\text{F}}$ is the tooth form factor, as defined in Eq. (29), ${\text{Y}}_{\text{S}}$ is the stress correction factor, as defined in Eq. (30), ${\text{K}}_{\text{A}}$ is the application factor, ${\text{K}}_{\text{V}}$ is the dynamic factor, ${\text{K}}_{\text{F}\beta }$ is the helix angle load distribution factor for bending strength calculation, ${\text{K}}_{\text{F}\alpha }$ is the face load distribution factor for bending strength calculation.

The expression for the tooth form factor is given as follows:


$$Y_{F}=\frac{\frac{6h_{Fe}}{m}cos\alpha_{Fen}}{{\left(\frac{S_{Fn}}{m}\right)}^{\text{2}}cos\alpha }$$ (29)

The stress correction factor is calculated using the following expression:


$$Y_{S}=\left(1.2+0.13L\right){q_{s}}^{\frac{\text{1}}{1.21+\frac{2.3}{L}}}$$ (30)

**Fig 10:**
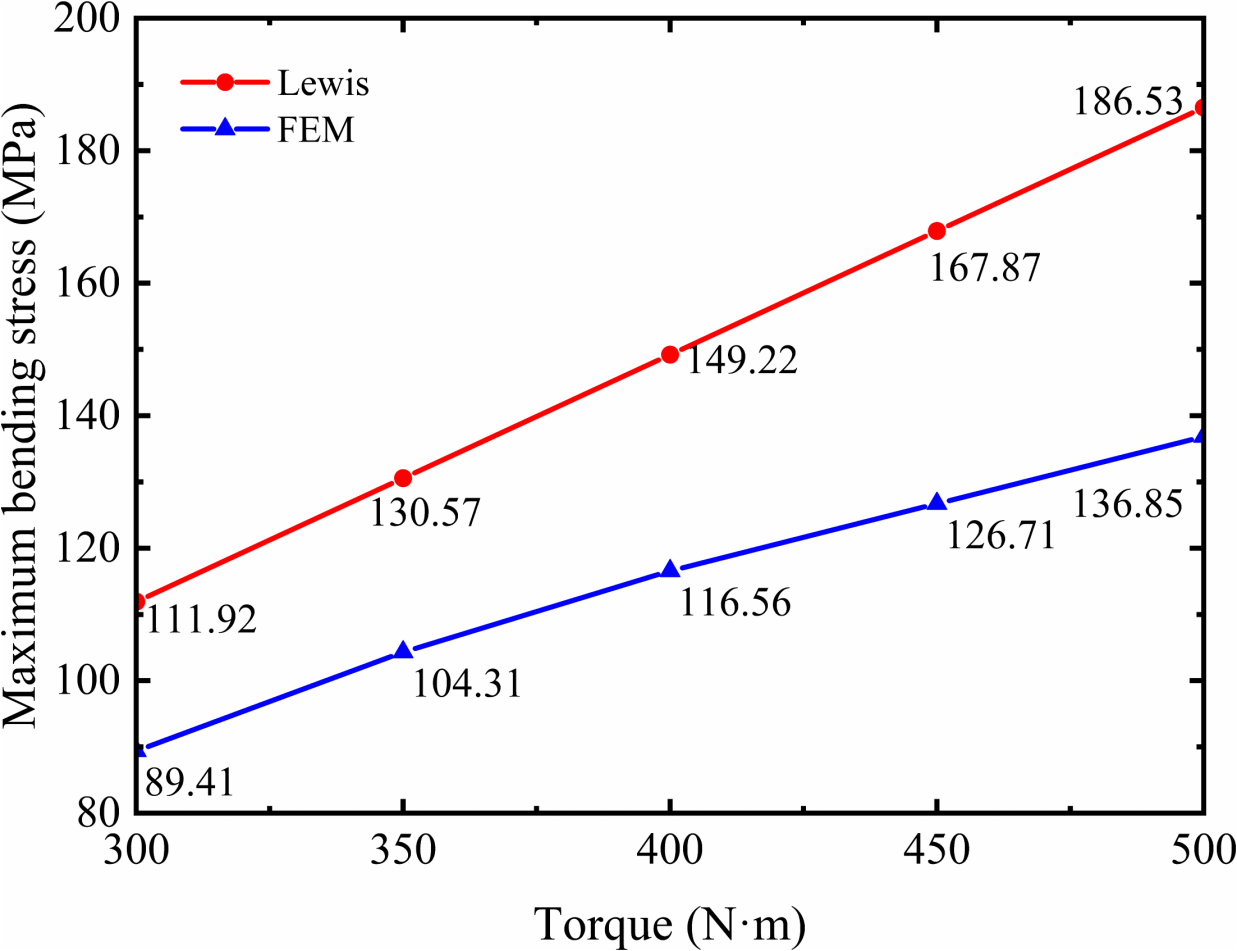
Comparison of Lewis and FEM maximum bending stress under varying torques.

The comparison between the maximum bending stresses calculated using the classical formula (Eq. (28)) under different torque levels and those obtained from finite element model is shown in Fig 10.

It can be observed that within the torque range of 300 N·m to 500 N·m, the variation curves of the maximum tooth root bending stress per meshing cycle—obtained from both the classical formula and the finite element method—are generally consistent in trend. However, the error increases gradually with torque, ranging from approximately 20.1% to 26.7%. The discrepancy is primarily attributed to the inherent limitations of the classical analytical formula and the use of the tensile-side stress at the tooth root. The Lewis formula is based on an approximation calculation using the cantilever beam assumption from material mechanics, which cannot effectively handle abrupt changes in the root section of gears, leading to inaccurate results in traditional gear bending stress calculations. By contrast, finite element analysis (FEA) offers a more precise and reliable means of evaluating tooth root stresses, as it captures the complex geometry and loading conditions more accurately. Thus, using FEA for gear bending strength assessment can effectively overcome the limitations of theoretical calculations and enhance the reliability of the results.

Through the validation of the strength models, it is observed that certain deviations exist between the results obtained from the finite element model and those derived from theoretical calculations. The discrepancy arises because the finite element model accounts for material properties and other design parameters, and more realistically simulates the gear meshing process. The stress variation trends of both models are largely consistent, confirming the accuracy and feasibility of using the finite element method for contact pressure and bending stress analysis in the study.

### 4.2 Comparative analysis of strength reliability between tip relief and standard gears

**Fig 11:**
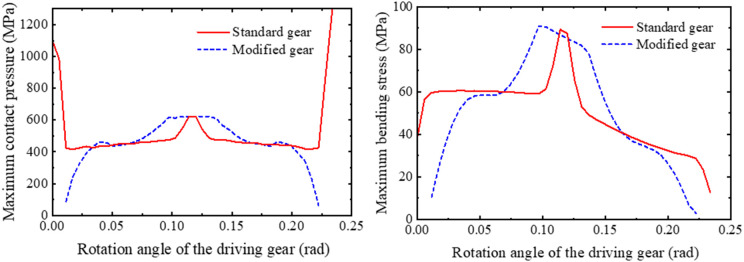
Comparison of stress distribution of gears.

**Fig 12:**
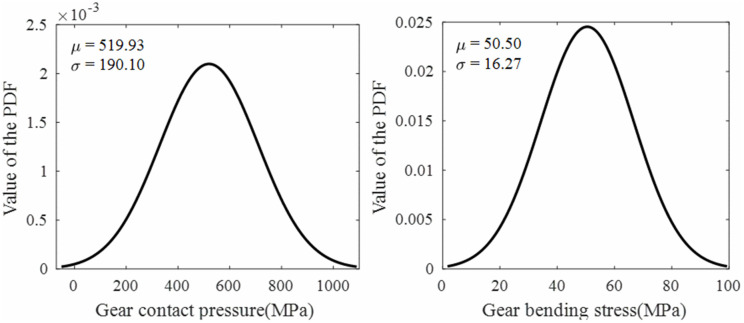
Fitted normal PDF of contact pressure and bending stress for standard gears.

**Fig 13:**
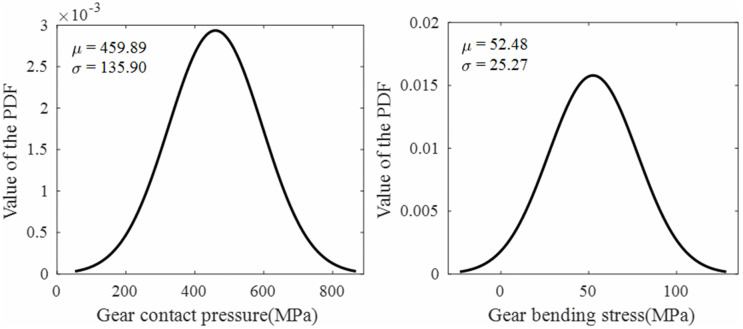
Fitted normal PDF of contact pressure and bending stress for tip relief gears.

Based on the strength models developed in Sec. 2, the stress distribution over a complete meshing cycle for a single tooth was obtained, as shown in Fig 11. When the rotation angle of the driving gear is 0 rad, it corresponds to the moment when the current meshing tooth pair begins contact at the start of the line of action (at the tooth root). The stress cycle covers a complete meshing cycle (single-tooth contact to double-tooth contact to single-tooth contact). MATLAB was used to calculate the mean and variance of both the contact pressure and bending stress of the gear, and to plot the probability density functions assuming a normal distribution. Import gear contact pressure and bending stress data within one meshing cycle into MATLAB for normal distribution fitting, resulting in the fitted normal probability density function (PDF) for gear contact pressure and root bending stress, as shown in Figs 12 and 13.

Through normal distribution fitting, it was determined that the contact pressure of the standard gear follows a normal distribution N(519.93, 190.10^2^), where the mean contact pressure ${\overset{―}{\mu }}_{\text{sH1}}$=519.93MPa and the standard deviation $\sigma_{\text{sH1}}$=190.10MPa. The bending stress of the standard gear follows a normal distribution N(50.50, 16.27^2^), with a mean of ${\overset{―}{\mu }}_{\text{sF1}}$=50.50 MPa and a standard deviation of $\sigma_{\text{SF1}}$=16.27 MPa. For the tip relief gear, the contact pressure follows a normal distribution N(459.89, 135.90^2^), with ${\overset{―}{\mu }}_{\text{sH2}}$=459.89 MPa and $\sigma_{\text{sH2}}$=135.90 MPa; the bending stress follows a normal distribution N(52.48, 25.27^2^), with ${\overset{―}{\mu }}_{\text{sF2}}$=52.48 MPa and $\sigma_{\text{sF2}}$=25.27 MPa.

The material used for the gears in the study is 40CrNiMoA, which has a hardness of 269 HB. Substituting the value into Eqs. (11) and (12), the calculated bending strength parameters are ${\overset{―}{\mu }}_{\text{rH}}$=673.49Mpa and $\sigma_{\text{rH}}$=37.66Mpa. Similarly, substituting the same hardness value into Eqs. (14) and (15), the calculated contact strength parameters are ${\overset{―}{\mu }}_{\text{rF}}$=917.29Mpa and $\sigma_{\text{rF}}$=37.66Mpa.

Using Eqs. (13) and (16), the standard gear contact strength reliability index is calculated as 0.79. Referring to the standard normal distribution table, the standard gear contact strength reliability is found to be 0.7852. The standard gear bending strength reliability index is 21.13, and from the standard normal distribution table, the standard gear bending strength reliability is approximately 1. For the modified gear, the contact strength reliability index is 1.51, and from the standard normal distribution table, the modified gear contact strength reliability is 0.93448. The modified gear bending strength reliability index is 19.07, and from the standard normal distribution table, the modified gear bending strength reliability is approximately 1.

Using Eqs. (17) and (18), the comprehensive reliability of the standard gear under combined contact and bending static strength failure modes was calculated as R_1_ = 0.79009. In contrast, the comprehensive reliability of the tip relief gear under the same failure modes was calculated as R_2_ = 0.94987. The corresponding reliability indices and calculated reliability values are summarized in Table 2.

**Table 2:** Reliability indices and values: standard vs. tip relief gears under different failure modes.

Item	Z_RH_	R_H_	Z_RF_	R_F_	Z	R
Standard gear	0.79	0.7852	21.13	1	0.79	0.79009
Modified gear	1.51	0.9345	19.07	1	1.51	0.94987

Note: Z_RH_ and R_H_ represent the reliability index and reliability, respectively, under the tooth surface contact strength failure mode. Z_RF_ and R_F_ represent the reliability index and reliability, respectively, under the tooth root bending strength failure mode. Z and R represent the comprehensive reliability index and overall reliability, respectively, considering multiple failure modes.

The data indicate that both the standard and tip relief gears exhibit high reliability under the combined contact and bending failure modes, reflecting a substantial safety margin. The reliability indices for bending strength are particularly high for both gear types, with their bending strength reliability values approaching 1. As a result, the comprehensive reliability index Z is primarily influenced by the reliability of contact strength. The suggests that, given the current material properties, the bending strength of the gears is sufficiently robust, and the overall reliability calculation can mainly focus on contact strength. However, under the same failure mode, the standard gear shows lower reliability compared to the tip relief gear. As shown in Fig 11, the contact pressure of the standard gear has obvious meshing impact at the engaging-in and recession point. Because in the transmission of straight gears without tooth top modification, when a pair of teeth contact at the tooth top and tooth root, due to factors such as tooth profile error and elastic deformation under load, the two tooth surfaces fail to achieve the theoretical smooth speed transition, resulting in an instantaneous impact force. This phenomenon is one of the main sources of gear vibration, noise, and additional dynamic load. It increases the mean and standard deviation of the contact pressure distribution leads to the decrease of the strength reliability of standard gear. After the tip relief, the meshing impact of the gear disappears and the reliability increases.

### 4.3 Effect of modification parameters on gear strength reliability

To further analyze the influence of tip relief parameters on the contact performance of gear tooth surfaces, based on the reshaping model mentioned in Section 2.1, a set of contact analyses was conducted on gear pairs with different modification configurations under a fixed modification index S=2. Both short-modification and long-modification cases were considered, and five data groups were selected for each variable. For consistency and simplification of the modeling process, the tooth profile parameters of the driving and driven gears were kept identical in all cases. In the short-modification scenario, the modification length of both gears was fixed at 1.2 mm. Five gear pairs (#1 to #5) were analyzed with varying modification height of 10 μm, 15 μm, 20 μm, 25 μm, and 30 μm, respectively. Next, to examine the effect of modification length, the modification height was held constant at 20 μm. Another five gear pairs (#6, #7, #3, #8, and #9) were analyzed with modification lengths of 1.0 mm, 1.1 mm, 1.2 mm, 1.3 mm, and 1.4 mm, respectively. In the long-modification scenario, the modification length for both gears was fixed at 2.6215 mm. Five gear pairs (#10 to #14) were examined with modification height of 5 μm, 10 μm, 15 μm, 20 μm, and 25 μm, respectively. In total, 14 different tip relief gear configurations were analyzed. The specific identifiers and corresponding modification parameters are summarized in Table 3.

**Table 3:** Modification parameters of spur gear pair.

Example id	Modification index S	Modification length L_t_/mm	Modification height Δ_m_/um
#1	2	L_t1_ = 1.2, L_t2_ = 1.2	Δ_m1_ = 10, Δ_m2_ = 10
#2	2	L_t1_ = 1.2, L_t2_ = 1.2	Δ_m1_ = 15, Δ_m2_ = 15
#3	2	L_t1_ = 1.2, L_t2_ = 1.2	Δ_m1_ = 20, Δ_m2_ = 20
#4	2	L_t1_ = 1.2, L_t2_ = 1.2	Δ_m1_ = 25, Δ_m2_ = 25
#5	2	L_t1_ = 1.2, L_t2_ = 1.2	Δ_m1_ = 30, Δ_m2_ = 30
#6	2	L_t1_ = 1.0, L_t2_ = 1.0	Δ_m1_ = 20, Δ_m2_ = 20
#7	2	L_t1_ = 1.1, L_t2_ = 1.1	Δ_m1_ = 20, Δ_m2_ = 20
#8	2	L_t1_ = 1.3, L_t2_ = 1.3	Δ_m1_ = 20, Δ_m2_ = 20
#9	2	L_t1_ = 1.4, L_t2_ = 1.4	Δ_m1_ = 20, Δ_m2_ = 20
#10	2	L_t1_ = 2.6215, L_t2_ = 2.6215	Δ_m1_ = 5, Δ_m2_ = 5
#11	2	L_t1_ = 2.6215, L_t2_ = 2.6215	Δ_m1_ = 10, Δ_m2_ = 10
#12	2	L_t1_ = 2.6215, L_t2_ = 2.6215	Δ_m1_ = 15, Δ_m2_ = 15
#13	2	L_t1_ = 2.6215, L_t2_ = 2.6215	Δ_m1_ = 20, Δ_m2_ = 20
#14	2	L_t1_ = 2.6215, L_t2_ = 2.6215	Δ_m1_ = 25, Δ_m2_ = 25

#### 4.3.1 Modification length.

**Fig 14:**
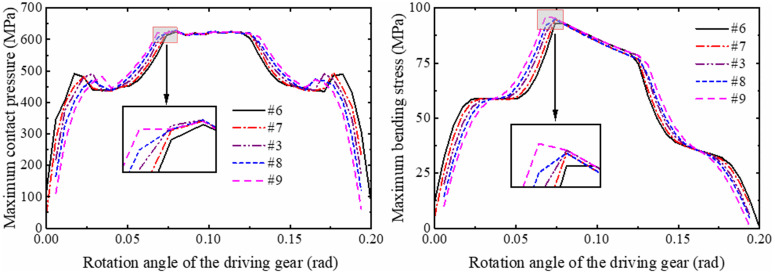
Stress distribution of gears with different modification lengths (20 μm modification height).

**Fig 15:**
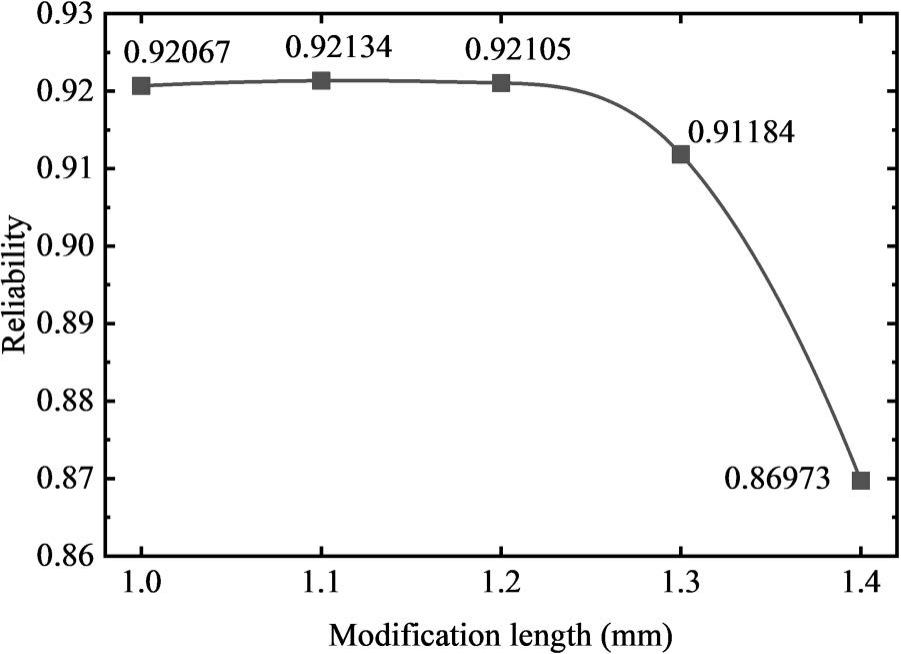
Reliability variation of gears with different modification lengths (20 μm height).

In order to obtain the change rule of the modification length on the reliability of the gear based on the contact strength and bending strength failure mode, the method of ensuring that other design variables remain unchanged and only changing the modification length of the gear is adopted. Five groups of gear models (#6, #7, #3, #8, #9) with modification lengths of 1 mm-1.4 mm were subjected to stress analysis of a meshing cycle, and the curves of contact stress and bending stress with rotation angle were obtained as shown in Fig 14. The normal fitting and reliability calculation are carried out, and the relationship between the strength reliability of the gear and the modification length is shown in Fig 15.

It can be observed that in Fig 15, with the modification height held constant at 20 μm, the reliability values of the gears with modification lengths of 1.0 mm, 1.1 mm, and 1.2 mm remain relatively stable, at 0.92067, 0.92134, and 0.92105, respectively. However, the reliability decreases to 0.91184 and 0.86973 for modification lengths of 1.3 mm and 1.4 mm, respectively. Overall, the results indicate a downward trend in reliability when the modification length exceeds 1.2 mm. As shown in Fig 14, it can be seen that under the condition of keeping the modification height unchanged, with the increase of the modification length, the meshing period gradually decreases, and the contact pressure and bending stress transition at the starting point of the modification are smoother. However, the gradual increase of the range of the single-tooth meshing area leads to the increase of the mean value of the contact pressure and bending stress of the tooth surface, which reduces the contact strength and bending strength of the gear, resulting in the reduction of the reliability of the gear.

#### 4.3.2 Modification height.

(1) Short-modification

**Fig 16:**
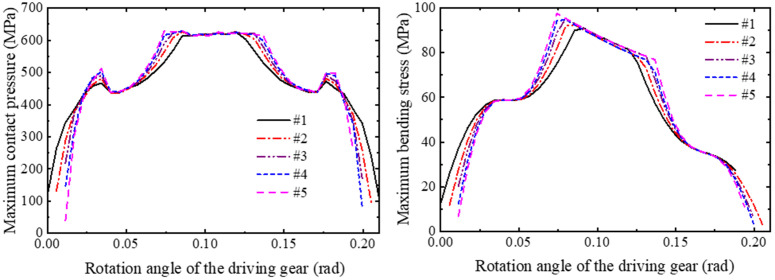
Stress distribution comparison: gears with different modification heights (short-modification).

**Fig 17:**
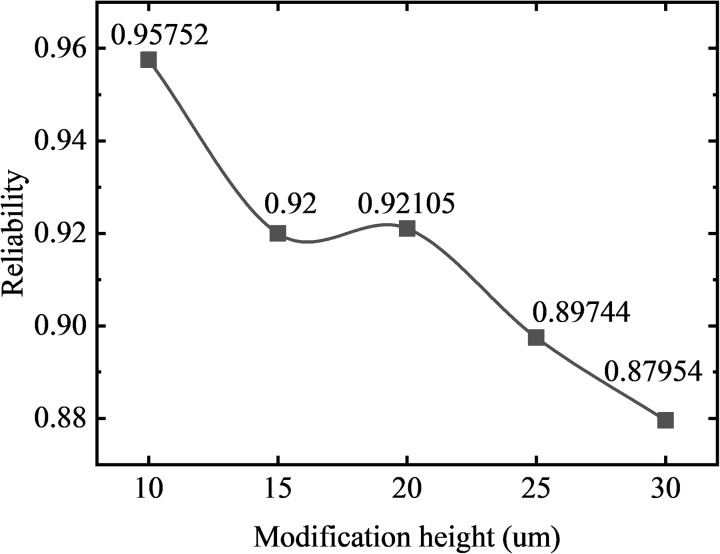
Reliability variation curves: gears with different modification heights (short-modification).

In order to obtain the variation law of the modification height on the reliability of the gear based on the contact strength and bending strength failure mode, the method of ensuring that other design variables are unchanged and only the modification height of the gear is changed. The stress analysis of five groups of gear models (#1, #2, #3, #4, #5) with short-modification length of 1.2 mm is carried out in one meshing cycle, and the curves of contact pressure and bending stress with rotation angle are obtained as shown in Fig 16. The relationship between the strength reliability of the short-modified gear and the modification height is obtained by normal fitting and reliability calculation as shown in Fig 17.

It can be observed that in Fig 17, under a fixed modification length of 1.2 mm, increasing the modification height from 10 μm to 30 μm results in a gradual decrease in gear reliability—from 0.95752 to 0.87954. The reliabilities for 15 μm and 20 μm modification height are 0.92000 and 0.92105 respectively, showing minimal difference. As shown in Fig 16, under the condition of keeping the modification length unchanged, with the increase of the modification height, the meshing period gradually decreases with the increase of the modification length, and the contact pressure mutation at the starting point of the modification gradually increases. At the same time, the gradual increase of the single-tooth meshing area leads to the increase of the mean value of the contact pressure and bending stress of the tooth surface, which reduces the contact strength and bending strength of the gear, resulting in the reduction of the reliability of the gear. Among them, the reliability of the gear group with the modification height of 20 um does not show a downward trend. Through the analysis of the stress change curve of the group of gears, it can be seen that although the average value of contact and bending stress increases, the stress values of the meshing point and the meshing point are larger, and the standard deviation of the normal distribution of stress decreases. The contact strength and bending strength of the gear remain basically unchanged.

(2) Long-modification

**Fig 18:**
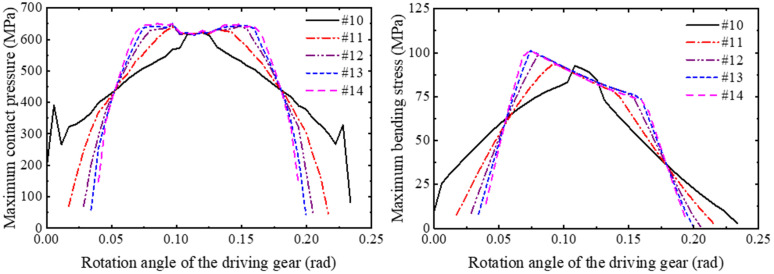
Stress distribution comparison: gears with different modification heights (long-modification).

**Fig 19:**
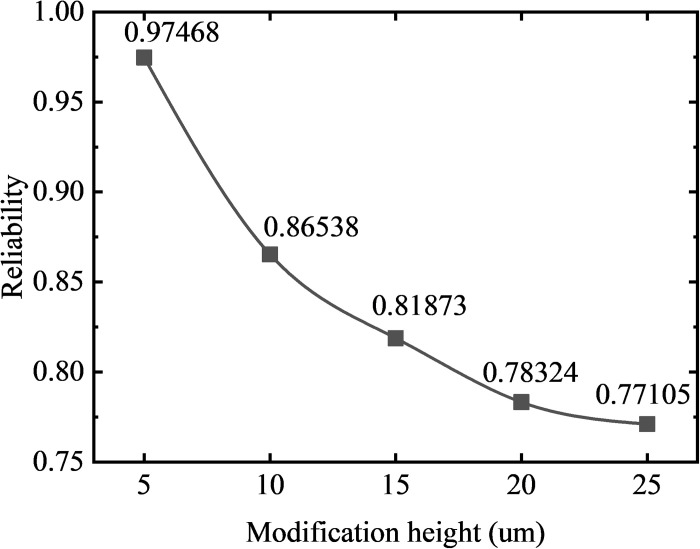
Reliability variation curves: gears with different modification heights (long-modification).

Similarly, the stress analysis of five groups of gear models (#10, #11, #12, #13, #14) with a modification length of 2.6215 mm is carried out in a meshing cycle, and the curves of contact pressure and bending stress with the rotation angle are obtained as shown in Fig 18. The normal fitting and reliability calculation are carried out to obtain the relationship between the strength reliability of the long-modified gear and the modification height as shown in Fig 19.

In Fig 19, it can be seen that under the condition of a fixed modification length of 2.6215 mm (long-modification), increasing the modification height from 5 μm to 25 μm leads to a more pronounced reliability drop—from 0.97468 to 0.77105. As shown in Fig 18, the long-modification gear group with the modification height of 5um has meshing impact at the engaging-in and recession point, and has stress mutation at the start point of the tip relief. With the increase of the modification height, the meshing period gradually decreases, and the range of the single-tooth meshing area gradually increases, which leads to the increase of the average contact pressure and bending stress of the tooth surface, reduces the contact strength and bending strength of the gear, and leads to the decrease of the reliability of the gear.

Compared with the short-modification, the gear under the condition of long-modification avoids the stress mutation at the start point of modification, but the strength reliability is more sensitive to the change of the modification height, and the smaller modification height cannot eliminate the meshing impact. Therefore, in the actual situation, the appropriate modification length and the modification height should be selected to ensure the strength reliability and meshing stability of the gear.

### 4.4 Effect of load on the strength reliability of tip relief gears

**Fig 20:**
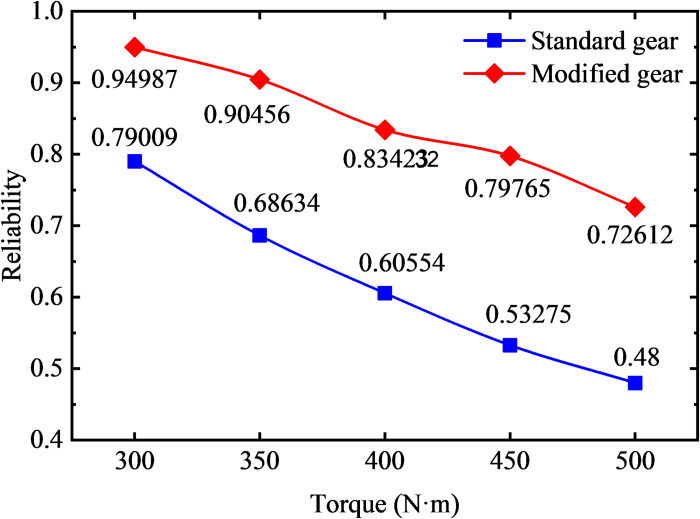
Curve of gear reliability under different loads.

**Fig 21:**
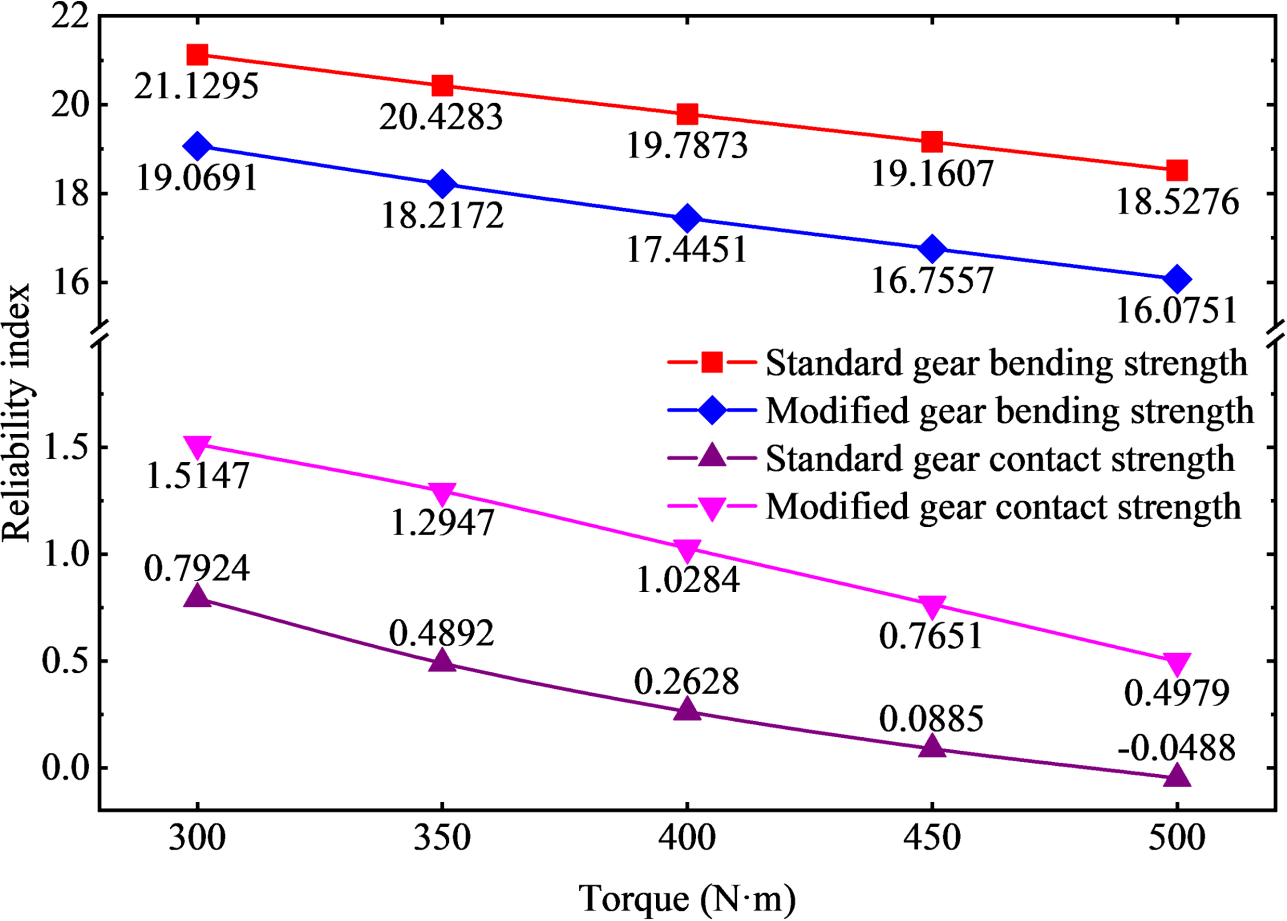
Curve of gear reliability index variation under different loads.

In order to explore the influence of different loads on the strength reliability of the modified gear, the gear model with the modified length of 1.2 mm and the modification height of 20um is selected and compared with the standard gear. The stress analysis of a meshing cycles is carried out by changing the load size, and the stress distribution curve is subjected to normal fitting and reliability calculation. The relationship between the reliability and reliability index of the standard gear and the modified gear with the load size is shown in Figs 20 and 21.

As shown in Fig 20, under torque loads ranging from 300 N·m to 500 N·m (in 50 N·m increments), the reliability of the standard gear model decreases progressively—from 0.79009 at 300 N·m to 0.48 at 500 N·m. And the reliability of the tip relief gears decreases gradually from 0.94987 at 300 N·m to 0.72612 at 500 N·m. It can be concluded that under unchanged conditions, increasing the load reduces the strength reliability of the gears based on contact and bending failure modes. Under the same load, the strength reliability of the tip relief gears is higher. The reliability of the contact strength of the standard gears is relatively low due to the great meshing impact. In practical engineering applications, especially under heavy load conditions, it is necessary to modify the addendum of the gear to eliminate the meshing impact and increase the reliability of the gear system. As shown in Fig 21, with the increase of load, the reliability index of contact strength and bending strength of gears decrease, among which the reliability index of contact strength is lower, and the contact strength is the main factor affecting the reliability of gears.

## 5. Conclusions

In the work, the strength reliability of the tip relief gears is investigated. Based on the finite element theory and the stress-strength interference theory, a reliability evaluation model is developed that accounts for both the contact and the bending strength failure modes. Furthermore, the effects of the modification parameters and the applied loads on the strength reliability of the gears are studied. The objective is to explore the influence of the tip relief on the gear strength reliability and to provide the theoretical support for improving the service life of the profile modified gears. The conclusions are as follows:

(1) The strength reliability of the gear pair increases after the tip relief of the tooth surface profile. Tooth tip relief can make the contact pressure distribution of the gear drive more uniform. The gear after tip modification eliminates the contact pressure impact of the engaging-in and recession point, reduces the mean and standard deviation of the gear contact stress, and increases the strength reliability of the gear.
(2) The strength reliability of the gears gradually decreases with the increase of the modification length when the modification height keeps a constant. The increase of the tip relief length leads to the stress mutation at the start point of the tip relief is smoother, but the increase of the single-tooth meshing area, resulting in the increase of the mean and standard deviation of the contact pressure and the bending stress, leading to the decrease of the gear’s strength reliability.
(3) The strength reliability of the gears gradually decreases with the increase of the modification height. The increase of the tip relief height leads to the stress mutation at the start point of the tip relief gradually increases. After long-modification, the stress transition during gear meshing becomes smoother, reducing the stress mutation at the start point of modification. The single-tooth meshing area increases when the modification height increases to a certain value, resulting in the increase of the mean and standard deviation of the contact pressure and the bending stress, leading to the decrease of the gear’s strength reliability.
(4) The strength reliability of the gears gradually decreases with the increase of the applied load. The reliability of the profile modified gears decreases at a faster rate because the contact ratio is reduced by the tip relief, which shortens the arc length of addendum circle and increases the single-tooth meshing area, thus lowering the overall gear strength and reliability.

In summary, the gear eliminates the meshing impact of the engaging-in and recession point after the tip relief, which have higher strength reliability under the same load conditions. The increase of the modification length can make the stress transition of the single-tooth and double-tooth meshing area of the gear smoother. The increase of the modification height under the condition of short-modification will increase the stress mutation at the starting point of the modification, but the long-modification can eliminate the mutation. As the modification length and modification height increase, the contact ratio of gear pair decreases further, leading to an extended period where a single pair of teeth bears all contact pressure and bending stresses, which results in a decline in gear strength reliability. In practical engineering applications, suitable modification parameters should be selected based on operational conditions to ensure strength reliability while reducing meshing impact and enhancing fatigue life. In addition, the finite element contact model and stress-strength interference reliability calculation method established in the paper can be extended to tooth direction modification and composite modification of tooth profile and tooth direction. The key lies in expanding the modification parameters from tooth profile parameters to parameters including tooth direction crowning amount, helix angle correction, etc., constructing finite element models for tooth direction modification and composite modification of tooth profile and tooth direction to output stress curves, and analyzing the impact of modification parameters on reliability.

## Supporting information

S1 File Data. (ZIP)
